# A 39 year-old woman with a mucinous cystadenocarcinoma of pancreas in the left upper abdomen and right lung: A case report

**DOI:** 10.1016/j.ijscr.2025.111813

**Published:** 2025-08-15

**Authors:** Arian Karimi Rouzbahani, Bahar Amiri, Behzad Yaghoubi, Sepideh Hadimaleki, Mohammad Kazem Shahmoradi, Ali Pajouhi

**Affiliations:** a Student Research Committee, Lorestan University of Medical Sciences, Khorramabad, Iran; b Department of Pathology, Imam Reza Hospital, Tabriz University of Medical Sciences, Tabriz, Iran; c Department of Surgery, Lorestan University of Medical Sciences, Khorramabad, Iran; d USERN Office, Lorestan University of Medical Sciences, Khorramabad, Iran

**Keywords:** Case report, Pancreas, Mucinous cystic neoplasm, Pulmonary mass, Tissue adhesions

## Abstract

**Introduction and importance:**

Mucinous cystic pancreatic neoplasms (MCPNs) are cystic lesions often seen in the body and tail of the pancreas.

**Case presentation:**

In the case reported here, we presented a 39-year-old woman with complaints of upper abdominal pain. It presents with a variety of signs and symptoms, the most common being upper abdominal pain, early satiety, and dyspnea.

**Clinical discussion:**

The diagnosis of MCPN was done postoperatively based on pathological and immunohistochemical studies. She underwent two operations: a midline laparotomy to remove the retroperitoneal mass, and a posterolateral thoracotomy to remove the pulmonary mass. To the best of our knowledge, this is the first reported case of mucinous cystadenocarcinoma of the pancreas in the left upper abdomen, invaded as a pulmonary mass in the right lung.

**Conclusion:**

MCPNs frequently appear in the pancreatic body and tail. They can also be found in the mediastinum.

## Highlights

• MCPNs are often seen in the body and tail of the pancreas.
• MCPNs impact women in their middle age, with a ratio of 20 females to 1 male.
• Surgical resection is the recommended treatment for symptomatic MCPNs.
• MCPNs can also be found in the mediastinum.

## 1 Introduction

Mucinous cystic pancreatic neoplasm (MCPN) is a type of tumor that originates from the pancreas and is characterized by the production of mucin and the formation of cysts [1]. Mucinous cystic pancreatic neoplasms predominantly impact women in their middle age, with a ratio of 20 females to 1 male. The average age at which these neoplasms are diagnosed is 48 years [2]. Nevertheless, as the majority of MCPNs do not show any symptoms, the actual occurrence and prevalence of MCPNs remain uncertain [2]. To assess a patient with a suspected MCPN, it is necessary to conduct several tests. These include a thorough examination of the blood, including a complete blood count and comprehensive metabolic panel. Additionally, a serum lipase test, a cancer antigen 19-9 (CA 19-9) test, and a carcinoembryonic antigen (CEA) level test should be performed. Imaging tests such as a triple-phase pancreas-protocol CT scan or magnetic resonance imaging (MRI) with contrast should be done to obtain detailed images of the pancreas. Furthermore, an endoscopic ultrasound with cyst wall biopsy and cyst fluid cytology should be conducted to gather more information. These tests are crucial for a comprehensive evaluation of the patient's condition [[2], [3], [4], [5]]. Surgical resection is the recommended treatment for MCPNs with high-risk characteristics or symptomatic lesions. It is generally advised that all mucin-producing neoplasms undergo resection due to their potential to become malignant [2,[6], [7], [8]].

In this particular case, we thoroughly examined the clinical, pathological, immunohistochemical, and laboratory features of a 39-year-old female patient who had mucinous cystadenocarcinoma of the pancreas. As far as we know, this is the initial documented instance of MCPN affecting the thorax and lungs.

## 2 Guideline citation

This case report has been reported in line with the SCARE criteria [9].

## 3 Timeline

She underwent a midline laparotomy to remove the retroperitoneal mass on March 4, 2023, and after less than 1 month, a splenectomy and a posterolateral thoracotomy to remove the pulmonary mass. After six months of assessment, an exploratory laparotomy was performed on October 6, 2023, and three cystic lesions filled with a mucinous-thick liquid were found in the retroperitoneum, pathologically confirmed as mucinous (Table 1).

**Table 1:** Timeline.

Date	Event description
September 2022	A 39-year-old female patient complains of upper abdominal pain. It presents with a variety of signs and symptoms, the most common being upper abdominal pain, early satiety, and dyspnea. This pain was worsened by meals and diminished with rest.
March 4, 2023	She underwent a midline laparotomy to remove the retroperitoneal mass.
April 2, 2023	A splenectomy and a posterolateral thoracotomy were performed to remove the pulmonary mass.
October 6, 2023	After six months of assessment, an exploratory laparotomy was performed, and three cystic lesions filled with a mucinous-thick liquid were found in the retroperitoneum, pathologically confirmed as mucinous.
December 2023	The follow-up after surgery indicated that the postoperative recovery was uneventful.

## 4 Patient information and clinical findings

A 39-year-old female patient was hospitalized on March 4, 2023, with complaints of upper abdominal pain. Her weight was 55 kg, her height was 157 cm, and her BMI was 22.05. It presents with a variety of signs and symptoms, the most common being upper abdominal pain, early satiety, and dyspnea. This pain was worsened by meals and diminished with rest. She had no history of trauma or other diseases and no notable medical history. She had vital signs with a pulse rate of 110 beats per minute, a blood pressure of 120/80 mmHg, and a respiratory rate of 36 min. On abdominal examination, a 30 cm × 30 cm mass in the left upper abdomen was found, extending up to the left subdiaphragmatic space, with a clear boundary and poor mobility. The abdomen was soft, and there was no tenderness. The other physical examinations were unremarkable.

## 5 Diagnostic assessment

**Fig. 1:**
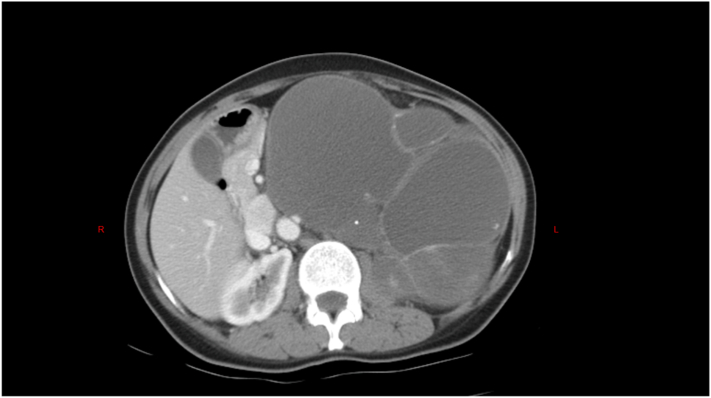
Computed tomography (CT) image of the large hypoechoic abdominal mass with cystic components posterior to the stomach and pancreas extending up to the left subdiaphragmatic and splenorenal spaces.

**Fig. 2:**
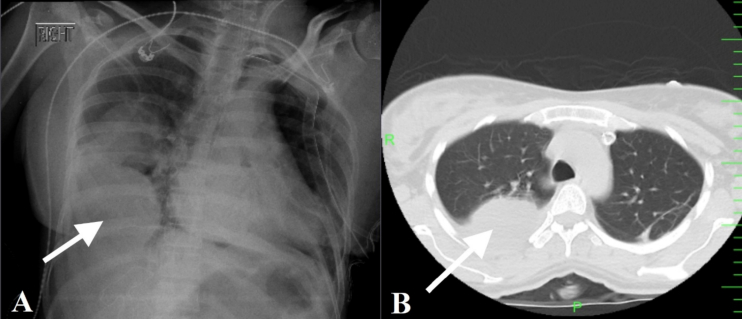
This figure shows a pulmonary mass in the lower middle segment of the right lung on chest radiograph (A) and CT scan (B).

Her complete blood count (CBC) showed microcytic, hypochromic anemia with an Hb of 7.8 g/dL and a normal platelet and white cell count. Her renal function and liver function tests were within normal limits. Her abdomen computed tomography (CT) revealed a large hypoechoic mass with cystic components posterior to the stomach and pancreas extending up to the left subdiaphragmatic and splenorenal spaces (Fig. 1). Chest radiographs and CT scan showed a pulmonary mass in the lower middle segment of the right lung (Fig. 2).

## 6 Therapeutic intervention

**Fig. 3:**
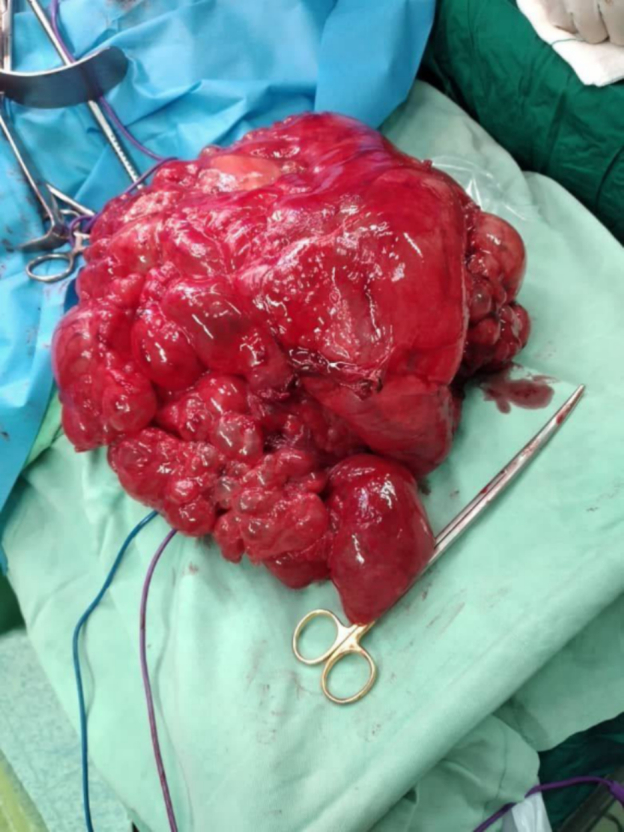
The abdominal mass right after the surgery.

The patient's treatment consisted of two stages of surgery. The first surgery was performed according to the standard technique with a midline incision. The surgical team consisted of a general surgeon, two general surgery residents, an anesthesiologist, a nurse anesthetist, and a surgical technologist. Under general anesthesia, after prep and drape, we entered the abdominal cavity in the supine position with a midline incision. There was a mass in the retroperitoneum with adhesions to the spleen, pancreas, aorta, and diaphragm. First, we entered the lesser sac space. Due to the adhesions of the adjacent organs, the mass was released. By cutting on the shaft of the mass, the gelatinous content was removed, which we suctioned. There were several small masses connected to the main mass, which were suctioned. In total, 4 L of gelatinous secretions were suctioned. The secretions were prevented from falling into the abdomen as much as possible. Due to the adhesion of the mass to the spleen, splenectomy was performed. The mass had numerous adhesions behind the pancreas and was released. On the left side, there was an adhesion to the diaphragm, which opened during the diaphragm release, and we repaired it with 1 proline. There were also adhesions to the vessels and aorta, which were also released, and the mass was completely removed, and samples were sent for pathological examinations (Fig. 3). Distal pancreatectomy was performed and oversewn with 0000 proline. No enlarged lymph nodes were observed in the retroperitoneum. After washing the abdomen, correctly counting the gases, and inserting a drain under the pancreas, the fascia and skin were sutured. The preoperative diagnosis was a hydatid cyst of the left lobe of the liver, but in pathological examinations, it turned out to be a mucinous adenocarcinoma of the pancreas. The duration of the operation was 3 h. After surgery, the patient was in NPO status for 48 h. After two days, a liquid-based diet was started, which the patient tolerated. Over the next 5 days, the diet was changed to regular, and the patient was discharged after tolerating PO. The drain was removed on the seventh postoperative day, and there was no discharge.

**Fig. 4:**
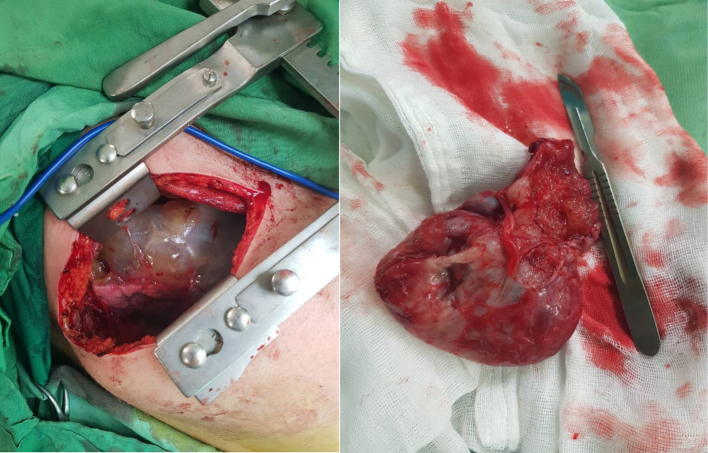
The pulmonary mass right after the surgery.

After less than 1 month, the patient was hospitalized again on March 28, 2023, due to her second surgery to remove the pulmonary mass. Under general anesthesia, after prep and drape, in the lateral position, an incision was made in the fifth intercostal space in the posterolateral part, and the skin and subcutaneous tissue were opened. The latissimus muscles were opened. The serratus muscles were retracted. The intercostal muscles were cut. We entered the plural space. A 15 cm diameter mass was observed in the interlobar region between the middle and lower lobes of the right lung with adhesions to the lung wall and umbilicus (Fig. 4). The adhesions were released. The mass contained mucinous secretions. Lavage was performed. The air leak site was repaired with Vicryl suture. A size 32 chest tube was inserted, and then the layers of the wall were sutured in anatomical order.

## 7 Follow-up and outcomes

**Fig. 5:**
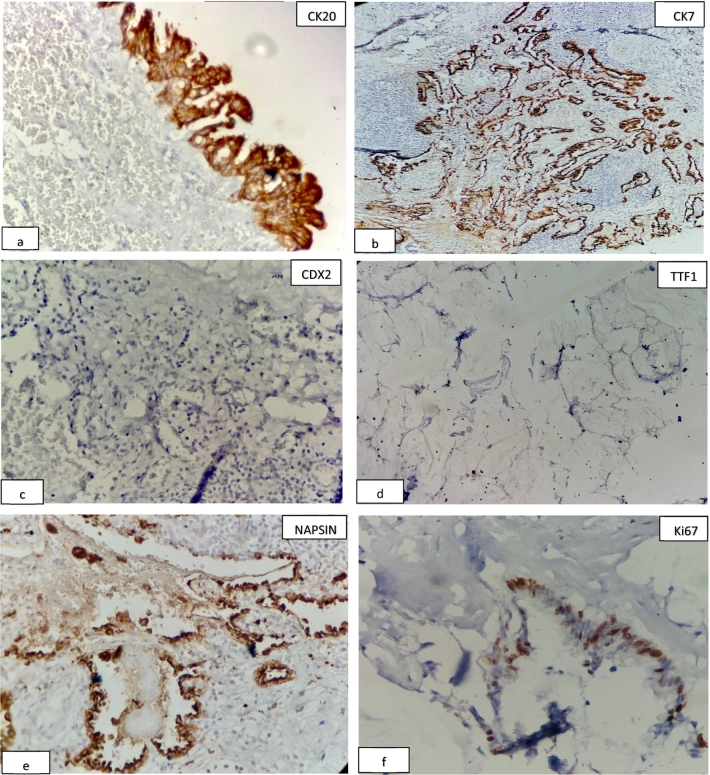
These IHC pattern are similar to mucinous tumor of lung. According to IHC results and history of the patient, final diagnosis in mucinous tumor of lung and peritoneum is from pancreatic origin. (a. b): cells with brown cytoplasmic staining for CK20 and CK7 are considered strong positive and indicated epithelial cell type origin (×40). (c, d): A negative staining in for CDX2 and TTF1 generally lowers the suspicion of a lung origin (×40). (e): Since napsin can be positive in few pancreatic mucinous tumors, the strong cytoplasmic positivity for napsin in our case, combined with findings from previous IHC, suggests a pancreatic origin for this tumor (×40). Ki67 index in tumoral cells are 50 % (×40) (f).

Pathological examinations showed a mucinous adenocarcinoma that originated from the pancreas. Mural nodules, thickening or irregularity of the walls, or calcification were not reported. Dysplastic cells were confined to one of the surgical margins. All surgical margins were free of tumor. The serum levels of the tumor markers carbohydrate antigen 19-9 (CA 19-9) and carcinoembryonic antigen (CEA) were normal, but CA 125 was 37.6 U/mL. Immunohistochemistry (IHC) results were in favor of a pancreatic mucinous neoplasm, with positivity for CK7, CK20, and napsin and a low level of TTF1 (Fig. 5).

**Fig. 6:**
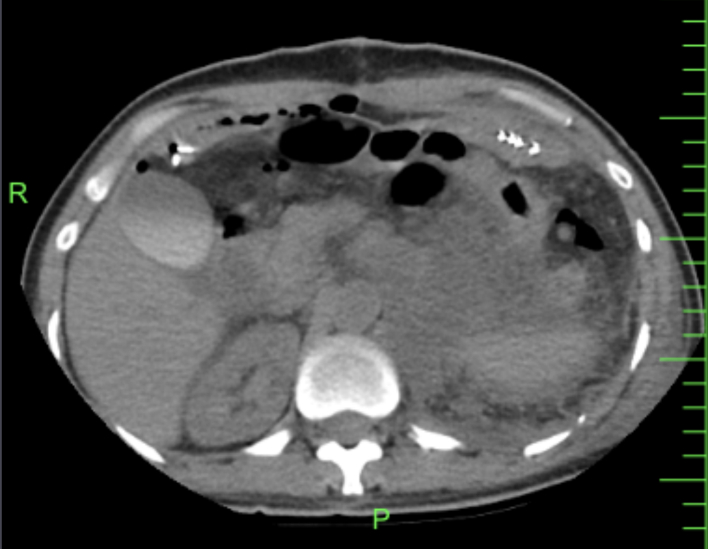
Follow-up CT scan images after the second laparotomy showing no residual mass.

After six months of assessment at Ashayer Hospital in October 2023, her primary complaint was worsening epigastric pain postprandially. A physical examination revealed moderate abdominal pain, with vital signs as follows: Temperature 36.8 °C, blood pressure 120/58 mmHg, heart rate 120 bpm, respiratory rate 37 bpm, and oxygen saturation 99 % in room air. The abdomen was soft, with tenderness in the epigastric area, while other systematic examinations were unremarkable. An exploratory laparotomy was performed on October 6, 2023, and three cystic lesions filled with a mucinous-thick liquid were found in the retroperitoneum, pathologically confirmed as mucinous adenocarcinoma of the pancreas. The subsequent postoperative recovery was uneventful (Fig. 6). The patient did not require admission to the intensive care unit, and she is alive.

## 8 Discussion

Mucinous cystic neoplasms (MCN) are rare, although their prevalence has increased due to developments in imaging techniques [10,11]. Mucinous cystic neoplasm (MCN) is a type of tumor characterized by the formation of cysts and induced by the proliferation of columnar epithelial cells. The illness has the capacity to develop into a malignant state, especially in patients with long-standing cases and large tumors [12]. Mucinous cystic pancreatic neoplasms (MCPNs) are cystic formations in the exocrine pancreas that secrete mucin. They frequently appear in the pancreatic body and tail [13].

Since the early 1990s, numerous publications have documented the presence of mucinous cystic neoplasms in the pancreas [6,7,[14], [15], [16], [17], [18]]. Reddy's research [18] revealed that the majority of patients with MCN were women (98 %). The neoplasms were predominantly located in the pancreatic body/tail area (93 %) and had a low occurrence of invasive malignancy upon resection (<10 %). Enlarged Metastatic Colorectal Peritoneal Nodules (MCPNs) can cause compression on adjacent organs and structures, leading to abdominal pain and a sensation of satiety. Occasionally, they can cause obstruction in the ducts and provoke acute inflammation of the pancreas. Typically, MCPNs in the pancreatic body and tail do not show any symptoms until they grow to a considerable size [19]. Most laboratory tests performed on people with MCN produce findings that fall within the normal range. Nevertheless, persons diagnosed with MCN and aggressive cancer frequently demonstrate increased levels of CA199. Previous studies have shown that elevated levels of carcinoma embryonic antigen (CEA) or CA199 are indicative of a greater probability of having a malignant condition [20].

In this case, CA 19-9 and CEA were normal, but CA 125 was 37.6 U/mL. Computed tomography (CT) is a commonly used imaging method that is essential for differentiating pancreatic mucinous cystic tumors from other medical problems. Our patient's CT scan revealed a large hypoechoic mass with cystic components posterior to the stomach and pancreas extending up to the left subdiaphragmatic and splenorenal spaces. Preoperative evaluations can be used as a diagnostic tool to anticipate the presence of pancreatic mucinous cystadenocarcinoma and pancreatic mucinous cystadenoma prior to surgery. Nevertheless, the diagnosis still requires confirmation through pathological tests. MCNs may contain both benign and malignant epithelial cells, which must be removed [21]. Given its malignant characteristics and its ability to withstand chemotherapy and radiotherapy, surgery is the preferred method of treatment for MCN [22]. Our patient underwent a midline laparotomy to remove the retroperitoneal mass, and after less than 1 month, a splenectomy and a posterolateral thoracotomy to remove the pulmonary mass.

In the case reported here, we conducted a detailed investigation of the clinical, pathological, immunohistochemical, and laboratory characteristics of a 39-year-old woman with mucinous cystadenocarcinoma of the pancreas. To the best of our knowledge, this is the first reported case of MCPN involving the thorax and lungs. MCPNs are cystic lesions often seen in the body and tail of the pancreas, but they can also be found in the thorax.

## Author contribution

B.A, A.K., A.P., S.H., M.S., and B.Y. contributed to the design and implementation of the research and to the writing of the manuscript.

## Consent for publication

Written informed consent for publication of their clinical details and clinical images was obtained from the patient. A copy of the consent form is available for review by the Editor of this journal.

## Ethical approval

This case report did not require ethical approval from Ethics Committee of Lorestan University of Medical Sciences. We have a written and signed consent to publish the information from the patient prior to submission. Our patient gave his consent for images and information about himself relating to the subject matter above to appear in the identified journal and associated publications.

## Guarantor

Mohammad Kazem Shahmoradi.

## Patient perspective

The patient was satisfied with his treatment, and the subsequent postoperative recovery was uneventful.

## Research registration number

Not applicable.

## Declaration of Generative AI and AI-assisted technologies in the writing process

We acknowledge that we have utilized AI tools such as ChatGPT and QuillBot for paraphrasing and enhancing the fluency of our writing. We are grateful for the assistance these tools provide in improving our text.

## Funding

Not applicable.

## Conflict of interest statement

The authors declare that they have no competing interests.

## Data Availability

The datasets used during the current study are available from the corresponding author on reasonable request.
